# An Ethical Perspective on the Democratization of Mental Health With Generative AI

**DOI:** 10.2196/58011

**Published:** 2024-10-17

**Authors:** Zohar Elyoseph, Tamar Gur, Yuval Haber, Tomer Simon, Tal Angert, Yuval Navon, Amir Tal, Oren Asman

**Affiliations:** 1 Department of Brain Sciences, Faculty of Medicine, Imperial College, Fulham Palace Rd, London, W6 8RF, United Kingdom, 44 547836088; 2 Faculty of Education, University of Haifa, Haifa, Israel; 3 The Adelson School of Entrepreneurship, Reichman University, Herzliya, Israel; 4 The PhD Program of Hermeneutics & Cultural Studies, Bar-Ilan University, Ramat Gan, Israel; 5 Microsoft Israel R&D Center, Tel Aviv, Israel; 6 Sagol School of Neuroscience, Tel Aviv University, Tel Aviv, Israel; 7 Samueli Initiative for Responsible AI in Medicine, Faculty of Medical and Health Sciences, Tel Aviv University, Tel Aviv, Israel; 8 Department of Nursing, Faculty of Medical and Health Sciences, Tel Aviv University, Tel Aviv, Israel

**Keywords:** ethics, generative artificial intelligence, generative AI, mental health, ChatGPT, large language model, LLM, digital mental health, machine learning, AI, technology, accessibility, knowledge, GenAI

## Abstract

Knowledge has become more open and accessible to a large audience with the “democratization of information” facilitated by technology. This paper provides a sociohistorical perspective for the theme issue “Responsible Design, Integration, and Use of Generative AI in Mental Health.” It evaluates ethical considerations in using generative artificial intelligence (GenAI) for the democratization of mental health knowledge and practice. It explores the historical context of democratizing information, transitioning from restricted access to widespread availability due to the internet, open-source movements, and most recently, GenAI technologies such as large language models. The paper highlights why GenAI technologies represent a new phase in the democratization movement, offering unparalleled access to highly advanced technology as well as information. In the realm of mental health, this requires delicate and nuanced ethical deliberation. Including GenAI in mental health may allow, among other things, improved accessibility to mental health care, personalized responses, and conceptual flexibility, and could facilitate a flattening of traditional hierarchies between health care providers and patients. At the same time, it also entails significant risks and challenges that must be carefully addressed. To navigate these complexities, the paper proposes a strategic questionnaire for assessing artificial intelligence–based mental health applications. This tool evaluates both the benefits and the risks, emphasizing the need for a balanced and ethical approach to GenAI integration in mental health. The paper calls for a cautious yet positive approach to GenAI in mental health, advocating for the active engagement of mental health professionals in guiding GenAI development. It emphasizes the importance of ensuring that GenAI advancements are not only technologically sound but also ethically grounded and patient-centered.

## Introduction

### The Democratization of Information: From Print to Artificial Intelligence–Generated Content

The democratization of information has been described as the process of making knowledge more accessible, inclusive, and transparent to a broad audience, often facilitated by technological advancements [1]. Over the past few centuries, a transformation has occurred in how knowledge is accessed, disseminated, and used. Historically, access to information and technology was often restricted to a privileged few—aristocrats, the church, academics, researchers, and professionals who had the means to gather and interpret data. The printing press served as an important milestone in the democratization of information. With the development of the steam locomotive (trains) in the 1800s, printed newspapers and journals that included news and ideas could be disseminated quickly and relatively cheaply across large distances. More recently, in the 1990s, when the internet became widely accessible, search engines enabled widespread and decentralized access to knowledge. Web 2.0, a participatory web with wiki platforms and other people-centric websites, later leveraged the web and engaged its users and elicited their collective intelligence [2]. This was followed by open-source movements that promoted sharing code and software frameworks freely, allowing developers globally to build upon and improve existing technologies. All these advancements led to an unprecedented amount of information freely accessible to billions. As technology continues to be developed and advanced, we argue that a new era in information democratization has begun in 2022 when various generative artificial intelligence (GenAI) platforms opened their platform for anyone with an internet connection to be used. The current phase of technology democratization marks a shift away from its exclusive use by computer scientists, researchers, and artificial intelligence (AI) professionals and toward reaching a broader audience with less expertise. Users now have more opportunities to actively participate in improving current technologies and may play a larger role in their advancement. GenAI technologies, such as large language models (LLMs) with visual and auditory elements, provide billions of people with direct access to cutting-edge technology, transcending the concept of “end users” and allowing them to perform tasks previously reserved for those with extensive computer science knowledge. Today, laypeople can use such technologies to create code, software, and GenAI models by expressing their desires in a natural language. These technologies drive the democratization of knowledge and technology by providing tailored, personalized, and on-demand information on a massive scale.

While the growing popularity of GenAI has undoubtedly aided the democratization of information, it also raises serious concerns about surveillance and control. Considering insights from Foucauldian theories, the widespread integration of GenAI into social discourse raises concerns about the potential abuse of authority and narrative manipulation. Furthermore, relying on GenAI-driven decision-making processes risks reinforcing existing power dynamics and marginalizing specific voices in society. As GenAI affects more aspects of our lives, it is crucial to critically evaluate its implications on privacy, autonomy, and the integrity of information dissemination. This paper provides a sociohistorical perspective for the theme issue on “Responsible Design, Integration, and Use of Generative AI in Mental Health.” It considers the ethics of using GenAI for the democratization of mental health knowledge and practice.

### Democratization of Knowledge in Mental Health: A Permanent Shift

Since the launch of ChatGPT (OpenAI) in November 2022, multiple studies have shown the transformative potential of GenAI in mental health [3-12]. This is crucial to recognize as we delve into the advantages and risks of GenAI in democratizing mental health knowledge. GenAI can address the global shortage of mental health professionals, reshape mental health care, advance diagnostic accuracy, improve treatment personalization, and enhance the overall accessibility of mental health services. It can facilitate mental health education and awareness, provide various self-help or self-paced mental health support tools, and so forth. However, it also poses risks, especially in the context of therapy and personalized mental health interventions.

## Advantages of GenAI in Mental Health Democratization

### Accessibility

A fundamental challenge in the mental health field is the limited access to mental health care both in developed and developing countries, as well as the disparities in access to mental health care [13-18]. Factors such as socioeconomic status [14], geographical location [19], linguistic barriers [20], and cultural disparities [21] present significant hurdles to the accessibility of mental health services. GenAI may be leveraged in mitigating these barriers through the development of linguistically and culturally attuned resources and potentially offering solutions adaptable to various economic backgrounds [19].

### Personalized Responses

AI provides an opportunity for a new era of mental health services that are sharply attuned to the individual needs and preferences of each patient [22]. Within the framework of treatment with a mental health professional, AI and GenAI technologies can facilitate a deeper understanding of a person’s unique psychological landscape by considering a myriad of factors such as their biological predispositions, societal and cultural influences, and personal preferences [23]. These technologies have the potential to analyze complex patterns and variances in individual experiences and histories, which can be instrumental in crafting responses and interventions that are tailored to suit individual needs as well as preferences. In contrast, non-AI–based search engines still used by many over the internet fall short of this goal, as searches often lack context and fail to grasp the nuanced complexities of individual experiences and histories. Moreover, personalized responses enabled by GenAI can envision a departure from generic diagnostic protocols, creating room for treatment paradigms that are designed with a more intricate understanding of individual idiosyncrasies. This method assumes that mental health is influenced by biological, social, and personal factors, aiming to provide solutions tailored to the distinct characteristics of each person’s experiences. Services that are tailored for each user specifically may lead the mental health field toward a more inclusive and equitable future.

### Theoretical or Conceptual Pluralism

In the pursuit of democratizing mental health, the goal is to enhance the field’s adaptability and inclusivity by embracing a variety of perspectives. GenAI mental health tools may be leveraged in three main ways and they are (1) providing a specific theory-based or perspective-based analysis of various scenarios with the ability to shift between theories or perspectives; (2) generating a dialogue or a synthesis between various theories or perspectives; and (3) potentially create a new theory or hypothesis (see Textbox 1).

Textbox 1. **Using generative artificial intelligence for multiple theory or perspective analysis, integration, and formation of novel ideas (generated by Claude AI 2.1 pro and ChatGPT 4; December 2023).**

Provide theory-based analysis

- Cognitive theory (Beck): The patient exhibits the key hallmarks of severe major depressive disorder per Beck’s cognitive model—pervasive negative cognitive schemas, automatized depressive cognitions, diminished mood, and inability to work.
- Object relations theory (Winnicott): The emergence of this severe depressive episode despite no clear environmental trigger suggests potential psychological vulnerabilities rooted in early childhood. Lack of good-enough mothering could have prevented the development of a cohesive self, resulting in a false self-presentation vulnerable to fragmentation and depressive affect under stress.

Synthesize perspectives

- This patient’s sudden-onset depression despite minimal stressors points to vulnerabilities in self-structure from poor early nurturing. His fragmented functioning aligns with Klein’s paranoid-schizoid position, while loss of values reflects ACT’s model. Synthesis suggests deficient parenting prevented integrating difficult experiences into a cohesive self. When his false self faltered, overwhelming feelings emerged that he could neither accept mindfully nor confront to rebuild his personality. Addressing both positive introjections in Kleinian play therapy and value-guided committed action using ACT may enable overcoming this acutely disabled state.

Create new theory

- The Wilted Plant Theory of Depression presents a unique botanical metaphor to understand and address depression. It likens the human psyche to a plant that requires “emotional sunlight” (positive interactions), “psychological nutrients” (intellectual engagement and meaningful activities), and “emotional hydration” (free expression of emotions) to thrive. Just as a plant wilts without proper care, the theory posits that the human mind endures in the absence of these essential elements, leading to depression. This metaphorical approach highlights the importance of a nurturing environment for maintaining and restoring mental well-being.

In other words, this pluralism may facilitate the synchronous operation of a variety of therapeutic approaches, philosophies, and cultural viewpoints. For instance, we can observe opportunities where GenAI might enable the integration and dialogue between traditionally distinct therapeutic methodologies such as cognitive behavioral therapy and psychodynamic approaches [24]. Here, the structural and goal-oriented strategies of cognitive behavioral therapy could be married with the depth of insight derived from psychodynamic explorations, engendering a more rounded approach to mental health care [25]. Moreover, the perspective of psychiatry, with its medically grounded insights, could be brought into conversation with psychological approaches, nurturing a space where medical, psychological, and holistic strategies can come together to form a more comprehensive view of mental health care.

With that being said, the way current LLMs work, the mere ability to be creative in connecting various methods in a convincing manner may be wonderful for brainstorming new eclectic concepts and therapeutic approaches but is in no way, in itself, evidence of its feasibility and reliability in real life.

### Equality and Reduction of Social Gaps

GenAI-powered LLMs hold the potential to foster greater equality and reduce prevailing social gaps [26]. By harnessing vast arrays of data and insights, such models may facilitate interventions crafted to meet the varied needs of different populations, including those historically underserved or marginalized [27,28]. For instance, developing and distributing mental health programs in languages and dialects that have historically lacked sufficient resources. It could further enable community-centric initiatives, enhancing the representation of diverse groups in the mental health discourse, thereby paving pathways for more localized and culturally sensitive interventions. Moreover, GenAI may be able to identify and relate to social aspects that are at times highly relevant in mental health scenarios. GenAI-based LLMs with access to information about symptoms, illnesses, and treatments, may allow laymen to ask questions and receive clarifications usually only available by contacting an expert. This may also facilitate in later contacting the relevant health care professionals, thus saving time and resources. This could become more relevant and useful when data sets used for training foundational models or fine-tuning general-purpose models have more representation of various languages and cultures.

### Therapist-Patient Engagement

One of the notable strengths of GenAI is its ability to reduce bureaucratic and administrative burdens in mental health care settings. It can provide transformative solutions by automating tasks like transcription, summarization [29], and form filling. Using these technologies, therapists may simplify administrative processes, freeing up more time and attention to provide direct patient care. With AI handling routine paperwork and data entry tasks, clinicians are freed from screens and forms, allowing them to focus on building a connection, conducting assessments, and providing personalized interventions to their patients. This not only increases the efficiency of mental health services, but also improves the overall quality of patient care by encouraging more meaningful interactions between therapists and their clients.

### Flattening of Hierarchies

The advent of GenAI bears the promising potential to flatten traditional hierarchical structures prevalent in the mental health sector, fundamentally altering the dynamics between health care providers and recipients [30]. Historically, psychiatrists and psychologists held a pronounced degree of authority, largely stemming from their exclusive access to specialized knowledge. If knowledge is no longer confined to a select few but is accessible to a wider population, this allows for a more balanced dynamic between mental health professionals and individuals seeking help [12,30]. It could empower individuals with insights and understanding of their own mental health conditions, fostering more collaborative therapeutic relationships and potentially leading to more fruitful and synergistic therapy sessions grounded on mutual understanding and shared knowledge. As we have previously defined, the introduction of GenAI into the field of mental health can be seen as an “artificial third” that changes the dynamic between mental health professionals and patients, so that in fact a new relationship triangle is created characterized by the flattening of the existing power hierarchy between experts and patients [9,12,31]. In this vision of a democratized mental health landscape, GenAI acts as an equalizer, breaking down barriers to knowledge accessibility and cultivating a health care landscape built on collaboration, understanding, and shared expertise. This is further evident with the context window of LLMs increasing dramatically over a short period of time (for instance, OpenAI ChatGPT increased from 4000 tokens to 128,000 on November 2023 and Google’s Gemini increased on February 2024 to 1 million tokens). This allows end users to upload a large amount of information (such as hundreds of text pages, images, and videos with clinically relevant information) and discuss it with a chatbot during one prompt or conversation.

## Risks to Mental Health Democratization Through AI

### Corporate Centralization

Corporate centralization in mental health services, facilitated by GenAI, carries a significant risk of prioritizing profit over individual-centered care, widening disparities in access and quality of mental health care, and influencing public health narratives toward economic gains rather than genuine support and care [32]. GenAI can assume a therapeutic role and be designed to foster trust and build rapport with users [33], making it a potent instrument in the hands of entities that may have their own agendas. This includes but is not limited to the promotion of specific political narratives, ideological indoctrination, aggressive marketing or unnoticeable marketing strategies, also known as dark pattern AI [34], taking advantage of its persuasive power for psychological manipulation and control. The centralization of power-knowledge, without emphasizing checks and balances in a small number of economic corporations could potentially create a facade of democratization of mental health, but not reflect a true democratization in the field.

### Information Transparency

Information transparency could be divided into 2 major aspects of the “one-way mirror,” as only 1 party is exposed to the other party’s information. On the provider side of the “mirror” there are real concerns about the management of user data. These include transactional misuses, such as unauthorized sales to third parties or its exploitation for targeted marketing [35]. However, more sinister breaches of personal privacy could also be achieved since GenAI systems have the potential to intrusively analyze personal conversations, behaviors, and emotions [5] without explicit consent. Moreover, the data harnessed might even be used in training AI systems, a process that remains largely concealed from the end users. Indeed, the algorithms driving this AI application function are much like a “black box” shrouded in mystery with no clarity as to how determinations and analyses are reached [36]. Alas, democratization is thus a double-edged sword; while GenAI may indeed democratize access to mental health resources, the current level of transparency and explainability to users of its operational mechanics may limit a truly informed user engagement, limiting the realization of a democratized system with empowered users [37]. When core aspects of an alignment process, including the embedded objectives, values, and ethics, are not made clear and transparent to users [7], it can result in power becoming concentrated in an entity whose true incentives remain obscured.

### People’s Misperceptions of AI

#### Overview

The level of expertise that people attribute to GenAI tools may be affected by their perceptions of technology. Numerous studies have shown that people tend to imbue AI systems with significant epistemic authority stemming largely from the veneer of impartiality and objectivity these technologies present. This attribution of high epistemic authority to GenAI systems may also pose a significant risk. Epistemic authority essentially refers to the weight and trust in a source as a repository of knowledge and information [38]. While GenAI systems can rely on a vast amount of data, the elevation of their epistemic authority could also carry detrimental effects for both health care providers and patients.

#### Risk of Misinformation

GenAI systems are not infallible; they can make mistakes, be based on incorrect data, or present biased viewpoints, thus generating incorrect advice or guidance. In the context of GenAI’s mistakes, consider mentioning the term “hallucinations” [39] or “confabulations” (which in our eyes is problematic terminology because it can be perceived as offensive and because it has an anthropomorphic assumption). Attributing high epistemic authority to GenAI may lead to unconditional acceptance of its output, without a critical evaluation of the veracity of the information provided.

#### GenAI Overreliance With Reduced Patient Self-Engagement

While incorporating GenAI into mental health care has numerous advantages, it also highlights the serious risk of epistemic bias among both therapists and patients. Attributing high epistemic authority to AI may overshadow not only the expertise and nuanced understanding of health care providers but also the personal experiences and insights of the patients themselves. Overreliance on GenAI in healthcare may reduce patient self-engagement by prioritizing AI-generated insights over the comprehensive understanding provided by health care providers and patients, potentially undermining individuals’ active participation in their mental health journey and resulting in less effective treatments. Relying on the AI’s ability to articulate and construct our own thoughts and feelings, thus “Letting the Machine speak for us” could also mean relinquishing effort in our interpersonal engagements, including in therapy, reducing one’s possibilities for self-understanding and growth [40]. Furthermore, therapists are vulnerable to epistemic bias by relying too heavily on AI-generated insights, potentially missing important nuances in patient narratives and clinical assessments. This overreliance on GenAI may unintentionally limit the therapist’s ability to engage deeply with patients, as algorithmic recommendations may not fully capture the complexities of individual experiences. As a result, it is critical for therapists to be vigilant against the influence of epistemic bias in their practice, striking a balance between using GenAI tools and retaining the essential human elements of empathy [41], intuition, and clinical judgment.

#### Increased Power Imbalance

The elevation of GenAI as a central epistemic figure may lead to a power imbalance, where knowledge is centralized in the hands of a GenAI entity that is under the control of economic corporations. This undermines the democratization ethos which seeks to foster a collaborative and pluralistic approach to mental health and where knowledge is the result of collective insight, involving a harmonic convergence of professional guidance and personal experiences. Thus, while GenAI offers a promise of democratized access to information, it also threatens to replace a current knowledge monopoly (currently in the hands of mental health experts) with a monopoly of a small number of LLM companies, which is counterintuitive to the principles of democratization that advocate for a decentralized, collective approach to knowledge dissemination and use. It should be noted that open-source models that are available to the public enable decentralized technological development and constitute a decentralized force, and as these models continue to develop and improve, the risks of a few companies monopolizing a field (including mental health) will be diminished.

People in emotional need may become dependent on or attached to GenAIs in potentially nonadaptive ways. For instance, many of Replika’s AI chatbot’s 7 million users see it as their best friend or even a family member [33]. While examining the relationship with this chatbot, researchers found that the patterns of dependency were different from other technological dependencies as it involved people feeling that Replika had needs and emotions that they needed to cater to [42]. Accordingly, there is an additional layer of risk relating to the authenticity of this “relationship” with a machine [5-8] whereby the humanization of GenAI [27] may imitate human agency in a manner that could alter our perception of good and healthy lives [43].

### Regulation Issues

As GenAI technology, driven mainly by for-profit private corporations, starts to enter the sphere of mental health services, there’s a growing concern regarding its adherence to the established protocols that have historically governed mental health services. While the democratization endeavor seeks to foster inclusivity and accessibility, the introduction of GenAI poses a conundrum; it opens avenues for unprecedented access to mental health resources but at the potential cost of diluting the standard of care and ethical considerations traditionally upheld by mental health professionals. One of the major bioethical and legal challenges in this regard is how care ethics concepts could be relevant within the developing field of “responsible AI,” to more fully consider AI’s impact on human relationships [44].

### Objective Perspective Versus Gender, Socioeconomic, and Ethnic Biases

Integrating GenAI in mental health services is challenged by how one balances between clinically informed judgments and reducing bias. AI systems rely on preexisting data that were produced, collected, and potentially also labeled by humans, thereby holding an intrinsic propensity to reflect societal biases, including those grounded in gender, socioeconomic factors, and ethnicity [45]. At the same time, demographic factors play a critical role in assessing individual health risks and conditions. Consequently, the AI alignment should continuously navigate the narrow pathway between eliminating biases and retaining critical data essential for accurate clinical judgments. From the democratization perspective, AI may perpetuate biases, and at the same time, if overly aligned, may fail to provide users’ expectations of a personalized and efficient mental health service. Thus, the path forward calls for a nuanced and vigilant development process for AI systems, one that meticulously harmonizes statistical evidence with fundamental democratic values.

The claims raised above suggest that AI represents a real opportunity to advance the field of mental health, as it will likely be increasingly present in our lives and its adoption seems inevitable. We propose addressing the risks outlined in this study as thought tools in the development of applied AI tools for responsible use in mental health, rather than viewing them as warnings against using this technology.

## Guiding Ethical Development: A Strategic Questionnaire for AI Mental Health Applications

Considering the potential risks and opportunities identified in the discourse on GenAI applications in mental health, we propose a set of carefully formulated questions designed to assess GenAI’s capability to enhance mental health care (Textbox 2). These questions are intended for use in the development processes of mental health applications, ensuring a comprehensive evaluation of both the benefits and the risks involved. We have deliberately distinguished between risks and opportunities, recognizing that they do not always exist on the same scale. Namely, a significant risk does not necessarily negate the potential benefits of an AI application, and vice versa. Hence, it is imperative to conduct a differential assessment for each application, weighing its specific risks against its potential opportunities. This approach is grounded in a nuanced understanding that while GenAI offers remarkable prospects for democratizing mental health care, its implementation must be navigated with caution to avoid unintended consequences. The proposed questionnaire is thus an extension of the discourse presented so far in the study, bridging the theoretical considerations with practical evaluation tools.

Textbox 2. **A strategic questionnaire for artificial intelligence mental health applications.**

Promoting democratization

- Accessibility: Does it improve access to mental health services for diverse individuals, including marginalized communities?
- User empowerment: Does it provide tools for self-care and informed decision-making?
- Facilitating collaboration and shared decision-making: Does it facilitate a collaborative approach between patients and health care providers, allowing for an AI augmented shared decision-making process?
- Inclusivity: Can it adapt to diverse cultural, socioeconomic, and personal needs promoting inclusive care?
- Transparency: Does it provide clear information about its functionalities, limitations, and data usage?

Identifying potential risks

- Data privacy and security: How are privacy and security risks mitigated?
- Bias and inequality: Does it reinforce societal biases or exacerbate inequalities in mental health care?
- Overdependence or addiction: How likely is it for users to develop over reliance or dependence on this tool?
  - Misinformation: How likely is the system to provide false or misinformation or lead to neglecting of human-based professional advice?
  - Corporate involvement: Are intentional or nonintentional considertations steering the clinical information or advice provided? Or to compromising ethical standards in patient care?
- Overshadowing human expertise: Does it diminish the role or undermine the expertise of mental health professionals?

## Discussion and Conclusion

The integration of GenAI in mental health care, as outlined in this paper, is a potent and inevitable aspect of the broader democratization movement. The ethical implications of not leveraging GenAI in this field are profound, given its potential to revolutionize care and treatment. GenAI introduces a paradigm shift, challenging existing dynamics within mental health care and presenting opportunities to resolve longstanding issues in the field. However, this shift is not without its challenges; it disrupts established power structures, provokes questions about truth and the nature of expertise, and raises concerns about the potential displacement of human roles by technology.

The transition to GenAI-driven mental health care is an inescapable reality, accompanied by considerable promises. It is imperative that the mental health field not only adapts to this new landscape but actively shapes it. This task should not be left solely to engineers and computer scientists; mental health professionals must play a pivotal role. Their involvement is critical to ensure that GenAI development aligns with the ethical standards and therapeutic goals of mental health care. In response to these challenges, our study proposes a structured questionnaire designed to guide responsible AI development in mental health. This questionnaire serves as a road map, delineating crucial considerations for balancing the opportunities and risks associated with GenAI integration. It emphasizes the need for a cautious yet optimistic approach to AI development and regulation, ensuring that advancements in mental health care are not only technologically sound but also ethically grounded and patient-centered. As we conclude, we call upon mental health associations and professionals to engage with these guidelines actively. By adopting a stance that is both critically vigilant and constructively engaged, the mental health field can navigate the complexities of GenAI integration. This approach is vital for harnessing AI’s potential while safeguarding the foundational values and ethical principles of mental health care. Our contribution, through this discussion and the questionnaire, aims to ensure that the AI revolution in mental health is not only technologically advanced but also democratically enriched and ethically sound.

## Abbreviations

AI: artificial intelligence
GenAI: generative artificial intelligence
LLM: large language model
